# Clinical performance of the multiplex solid-phase “Direct Strip PCR” for infectious uveitis: a multicenter diagnostic accuracy study

**DOI:** 10.1007/s10384-026-01343-2

**Published:** 2026-04-02

**Authors:** Satoko Nakano, Sunao Sugita, Hiroshi Takase, Yasuhiro Tomaru, Daisuke Todokoro, Yumi Hasegawa, Yasuhiro Ikeda, Go Mawatari, Chie Sotozono, Kenji Nagata, Kaoru Araki-Sasaki, Suguru Nakagawa, Yoshiaki Kiuchi, Kaori Mitoma, Nobuyo Yawata, Takehiro Hariya, Eiichi Uchio, Tomoko Tsukahara-Kawamura, Hidenori Inoue, Koji Toriyama, Sentaro Kusuhara, Wataru Matsumiya, Nobuyuki Ohguro, Hisashi Mashimo, Ken Fukuda, Waka Ishida, Mariko Egawa, Kei Nakai, Masakazu Morioka, Atsushi Hayashi, Tomoko Nakamura, Yoshihiko Usui, Hiroshi Goto, Ryohei Nejima, Kazunori Miyata, Kenichi Namba, Keitaro Hase, Annabelle A. Okada, Hiroshi Keino, Nobuhisa Mizuki, Masaki Takeuchi, Dai Miyazaki, Kunihiro Kiyosaki, Reiko Kinouchi, Ryoji Yanai, Sho-Hei Uchi, Shigeko Yashiro, Rie Tanaka, Aika Tsutsui, Takashi Suzuki, Yukiko Umeki, Takaaki Yahiro, Akane Sonezaki, Takashi Matsumoto, Tetsuji Ohyama, Masae Kuranari, Naoto Uemura, Yuichi Hori, Atsunobu Takeda, Toshiaki Kubota, Toshikatsu Kaburaki, Koh-Hei Sonoda, Manabu Mochizuki, the Direct Strip PCR Project Groups

**Affiliations:** 1 Department of Ophthalmology, Oita University Faculty of Medicine, 1-1 Idaigaoka, Hasama-cho, Yufu, Oita 879-5593 Japan; 2 Sugita Eye Clinic, Miyazaki, Japan; 3 Department of Ophthalmology, Kobe City Eye Hospital, Kobe, Japan; 4 Miyata Eye Hospital Tokyo Clinic, Tokyo, Japan; 5 Department of Ophthalmology, Gunma University Graduate School of Medicine, Maebashi, Japan; 6 Department of Ophthalmology, Faculty of Medicine, University of Tsukuba, Tsukuba, Japan; 7 Department of Ophthalmology, Faculty of Medicine, University of Miyazaki, Miyazaki, Japan; 8 Department of Ophthalmology, Kyoto Prefectural University of Medicine, Kyoto, Japan; 9 Department of Ophthalmology, Kansai Medical University, Hirakata, Japan; 10 Department of Ophthalmology, Saitama Medical Center, Jichi Medical University, Saitama, Japan; 11 Department of Ophthalmology and Visual Science, Graduate School of Biomedical and Health Sciences, Hiroshima University, Hiroshima, Japan; 12 Graduate School of Medical Sciences, Ocular Pathology and Imaging Science, Kyushu University, Fukuoka, Japan; 13 Department of Ophthalmology, Tohoku University Graduate School of Medicine, Sendai, Japan; 14 Department of Ophthalmology, Fukuoka University School of Medicine, Fukuoka, Japan; 15 Department of Ophthalmology, Ehime University School of Medicine, Toon, Japan; 16 Department of Ophthalmology, Graduate School of Medicine, Kobe University, Kobe, Japan; 17 Department of Ophthalmology, Japan Community Health Care Organization Osaka Hospital, Osaka, Japan; 18 Department of Ophthalmology and Visual Science, Kochi Medical School, Kochi University, Nankoku, Japan; 19 Department of Ophthalmology, Institute of Biomedical Sciences, Tokushima University Graduate School, Tokushima, Japan; 20 Department of Ophthalmology, Yodogawa Christian Hospital, Osaka, Japan; 21 Department of Ophthalmology, Faculty of Medical Sciences, University of Fukui, Eiheiji, Japan; 22 Department of Ophthalmology, Graduate School of Medicine and Pharmaceutical Sciences, University of Toyama, Toyama, Japan; 23 Department of Ophthalmology, Tokyo Medical University, Tokyo, Japan; 24 Miyata Eye Hospital, Miyakonojo, Japan; 25 Department of Ophthalmology, Faculty of Medicine and Graduate School of Medicine, Hokkaido University, Sapporo, Japan; 26 Department of Ophthalmology, School of Medicine, Kyorin University, Mitaka, Japan; 27 Department of Ophthalmology, Yokohama City University Graduate School of Medicine, Yokohama, Japan; 28 Division of Ophthalmology and Visual Science, Faculty of Medicine, Tottori University, Yonago, Japan; 29 Beppu Medical Center, Beppu, Japan; 30 Department of Ophthalmology, Asahikawa Medical University, Asahikawa, Japan; 31 Department of Ophthalmology, Graduate School of Medicine, Yamaguchi University, Ube, Japan; 32 Department of Ophthalmology, National Center for Global Health and Medicine, Japan Institute for Health Security, Tokyo, Japan; 33 Department of Ophthalmology, University of Tokyo, Tokyo, Japan; 34 Department of Ophthalmology, Shimane University Faculty of Medicine, Izumo, Japan; 35 Department of Ophthalmology, Toho University Faculty of Medicine, Tokyo, Japan; 36 Ishizuchi Eye Clinic, Niihama, Japan; 37 Department of Microbiology, Oita University Faculty of Medicine, Yufu, Japan; 38 Department of Environmental and Preventive Medicine, Oita University Faculty of Medicine, Yufu, Japan; 39 Biostatistics Center, Kurume University, Kurume, Japan; 40 Hoaki Hospital, Oita, Japan; 41 General Clinical Research Center, Oita University Hospital, Yufu, Japan; 42 Department of Clinical Pharmacology and Therapeutics, Oita University Faculty of Medicine, Yufu, Japan; 43 Department of Ophthalmology, Jichi Medical University, Shimotsuke, Japan; 44 Department of Ophthalmology, Graduate School of Medical Sciences, Kyushu University, Fukuoka, Japan; 45 Department of Ophthalmology & Visual Science, Graduate School of Medical and Dental Sciences, Institute of Science Tokyo, Tokyo, Japan

**Keywords:** Clinical performance study, Direct Strip PCR, Herpetic uveitis, Infectious uveitis, Multiplex PCR

## Abstract

**Purpose:**

To evaluate the clinical performance of “Direct Strip PCR,” a multiplex solid-phase real-time polymerase chain reaction (PCR) kit, for diagnosing infectious uveitis.

**Study design:**

Multicenter, prospective, diagnostic accuracy study

**Methods:**

We analyzed 475 ocular-fluid samples (336 aqueous humor and 139 vitreous fluid) from 29 sites in Japan. Direct Strip PCR, an IVD-grade multiplex real-time PCR kit targeting HSV-1, HSV-2, VZV, EBV, CMV, HHV-6, HTLV-1, *Toxoplasma gondii*, and *Treponema pallidum* requiring no DNA extraction and incorporating internal controls, was compared with quantitative PCR (qPCR) for concordance and DNA copy-number correlation.

**Results:**

All 9 of the target pathogens were detected. Direct Strip PCR showed excellent agreement with qPCR, with percent positive agreement (PPA), percent negative agreement (PNA), and percent overall agreement (POA) in aqueous humor of 98.0%, 99.2%, and 98.5%, respectively, and in vitreous fluid of 95.7%, 97.1%, and 96.4%, respectively, indicating high concordance for positive and negative results. All the values exceeded predefined 90% thresholds agreed upon with the Pharmaceuticals and Medical Devices Agency, meeting the required performance criteria. Discordant results were infrequent (10/4275 targets) and involved low-copy samples near the detection limit. The DNA copy numbers correlated strongly between the methods (r = 0.948–0.996). No adverse events were reported.

**Conclusion:**

Direct Strip PCR demonstrated high concordance with qPCR, reliably detected major pathogens of infectious uveitis, and yielded quantitative results that correlated with the qPCR results, supporting disease monitoring. Its solid-phase, extraction-free, and per-run calibration-free format provides a practical basis for regulatory submissions and global dissemination of multiplex PCR.

**Supplementary Information:**

The online version contains supplementary material available at 10.1007/s10384-026-01343-2.

## Introduction

Uveitis is an intraocular inflammatory disorder affecting the iris, ciliary body, and choroid, comprising approximately 40 subtypes, and posing significant diagnostic challenges [1, 2]. Causes of uveitis can be both infectious and noninfectious. Infectious uveitis, accounting for 15.4% of all cases in Japan, is caused by viruses, particularly human herpesviruses, parasites such as *Toxoplasma gondii*, bacteria, and fungi [1]. As each requires specific treatment, delayed diagnosis may cause vision loss and impact quality of life and health care systems.

Serologic antibody tests have limited diagnostic value owing to subclinical and localized infections. Since the 1990s, polymerase chain reaction (PCR) testing of aqueous humor (AH) and vitreous fluid (VF) has been widely adopted and is now performed at approximately 80% of the major institutions in Japan [3]. Intraocular pathogen load correlates with disease activity and prognosis [4–7], establishing quantitative real-time PCR (qPCR), including multiplex PCR for multiple pathogens, as the diagnostic gold standard. Multiplex PCR is incorporated into several diagnostic criteria, such as cytomegalovirus (CMV) endotheliitis (CMV-positive, herpes simplex virus [HSV]-negative, varicella-zoster virus [VZV]-negative) [8] and acute retinal necrosis (ARN; HSV-1-/HSV-2/VZV-positive) [9]. A nationwide survey involving 66 major uveitis centers in Japan reported that 100% of ARNs were diagnosed via PCR [1].

The SUN Working Group Classification Criteria in the United States list PCR positivity as a major criterion for HSV anterior uveitis (HSV-AU), VZV-AU, CMV-AU, ARN, CMV retinitis, and toxoplasmic retinitis [10–14]. For CMV infections, PCR is reported to be the most reliable standalone diagnostic method, showing the highest diagnostic performance with an AUC of 0.98; no single clinical sign achieves comparable accuracy [15]. Additionally, the Japanese Ocular Inflammation Society (JOIS) guidelines have emphasized the exclusion of infectious uveitis before initiation of immunosuppressive therapy for noninfectious cases [16]. Consequently, comprehensive and practical diagnostic methods for routine clinical use are warranted.

Intraocular fluid PCR testing for herpesviruses (HSV-1 to human herpesvirus [HHV]-8) [17], along with bacterial and fungal rDNA, was first approved under Advanced Medical Care, a Japanese framework for uninsured advanced medical technologies. On the basis of this framework, “Direct Strip PCR” for infectious uveitis [18–20]—a multiplex solid-phase real-time PCR kit without DNA extraction—was subsequently developed; it is used in Advanced Medical Care and outsourced diagnostic laboratories and is currently available at more than 160 facilities nationwide. It simultaneously detects 9 major pathogens—HSV-1, HSV-2, VZV, CMV, Epstein–Barr virus (EBV), HHV-6, human T-lymphotropic virus type 1 (HTLV-1), *Toxoplasma gondii*, and *Treponema pallidum*—from only 20 µL of intraocular fluid. These targets were optimized and validated through multicenter studies involving 772 and 511 samples [19, 20], selected from an initial set of 24 candidate targets [18] identified in previous reports using single real-time, multiplex [17] and broad-range PCR. Subsequent comprehensive analyses using metagenomic next-generation sequencing (NGS) have not identified any additional major pathogens [21, 22].

The Direct Strip PCR kit demonstrated complete analytical specificity for ocular infectious pathogens using target-sequence-specific TaqMan real-time PCR, while excluding environmentally persistent agents such as bacteria, fungi, and adenoviruses, thereby avoiding contamination issues associated with gel-based or SYBR Green PCR. Moreover, its solid-phase format eliminates nucleic acid extraction or precise reagent handling requirements, enabling rapid and consistent operation (1-min preparation, 32-min PCR). It achieved a limit of detection comparable to that of conventional qPCR, with a median of 12.0 copies/reaction. It is compatible with standard PCR instruments and provides stable quantitative results with minimal interuser and interlot variability (coefficient of variation [CV] <1.8%), even for beginners, making it highly suitable for clinical use [20]. In a multinational study across 18 sites and 511 samples, Direct Strip PCR demonstrated excellent concordance with qPCR (positive agreement: 98.8%–100%, negative agreement: 99.8%–100%, Cohen kappa [κ] = 0.969–1.000, *P* <.001–.031) [20]. Under the Advanced Medical Care program, intraocular fluid PCR testing for herpesviruses achieved 99.8% effectiveness with no adverse events and was appropriately applied to indicated cases [3].

Infectious uveitis is rare and has low commercial potential: national surveys report 2 to 94 new cases annually for each subtype [1] and an average of 24.8 tests annually per Advanced Medical Care facility, creating barriers to industry-led regulatory submission. Because of cost-related barriers to industry-led regulatory submission, IVD-equivalent products manufactured in QMS-compliant facilities were unavailable for an extended period, necessitating studies using research-use reagents (RUO) [18–20]. These efforts have led to a recommendation by the Japanese Ocular Inflammation Society (JOIS), designated under the Ministry of Health, Labour and Welfare’s “High Medical Need Device” program, and subsequent support from the Agency for Medical Research and Development (AMED) Translational Research Program (Seeds preB and F), which enabled the initiation of IVD/QMS-grade manufacturing by industry.

In this IVD/QMS-grade study, the PCR program was optimized by increasing the denaturation step to 98 °C for 10 s to mitigate the effects of higher-order DNA structures and to ensure robust performance across multiplex PCR platforms. The study was conducted under centralized double blinding with prospectively defined PMDA performance thresholds. Furthermore, both per-run 3-point calibration and representative single-run calibration were systematically evaluated to assess feasibility for routine clinical use. Direct Strip PCR was evaluated against qPCR for positive, negative, and overall concordance and copy-number correlation to support regulatory approval as an IVD in Japan and internationally.

## Methods

### Study design

This multicenter, prospective, physician-initiated diagnostic accuracy study was conducted at 29 ophthalmology centers across Japan.

### Study population

The inclusion criteria comprised AH or VF collected during routine examinations or surgeries from cases of infectious uveitis (HSV-1, HSV-2, VZV, EBV, CMV, HHV-6, HTLV-1, *T pallidum*, and *T gondii*), noninfectious uveitis, or nonuveitis such as cataract, epiretinal membrane, macular hole, or rhegmatogenous retinal detachment. The exclusion criteria included investigator-assessed ineligibility, insufficient volume (≤20 µL), or contamination with blood or solids. Given the rarity of the disease, the Pharmaceuticals and Medical Devices Agency (PMDA) allowed the use of cryopreserved samples from a previous study [20]; however, most had been consumed, leaving only 5 AH and 9 VF samples available, with 94.0%–98.7% newly collected. The study endpoints were (1) ≥30 qPCR-positive samples across all pathogens and (2) ≥50 qPCR-negative samples for all 9 pathogens, on the basis of IVD approval standards of 150 positive and 150 negative samples, with reduced numbers accepted owing to disease rarity and feasibility, as confirmed in previous PMDA consultations. The study adhered to the Declaration of Helsinki. It was approved by the ethics committee of Oita University on February 24, 2023 (with additional approvals from all the participating institutions), registered in the UMIN Clinical Trials Registry on May 10, 2023 (UMIN000050986), and conducted with written or optout consent until May 15, 2024. Written informed consent was obtained from all the participants or was obtained through an approved optout process, in accordance with institutional ethics regulations.

### Protocol procedures

Because intraocular fluids are rarely tested immediately after collection in hospital and external laboratory centers, the package insert specifies cryopreservation to prevent nucleic-acid degradation; this procedure was followed in this study, with samples transported on dry ice and stored in temperature-monitored freezers. Samples were diluted up to 5-fold to both address limited sample volume and comply with PMDA guidance requiring evaluation under stringent conditions, including low-copy specimens near the limit of detection (LoD). As indicated in the MIQE guidelines for real-time PCR experiments [23], moderate cryopreservation and dilution do not substantially affect quantitative PCR performance. The PMDA allowed such cryopreservation and dilution on the basis of validation data from an industry-initiated performance study; paired analyses showed strong correlations in Cq values before and after cryopreservation, including transportation (Pearson correlation coefficient: AH, r = 0.879; VF, r = 0.819; *P* <.001 for both); spike-in experiments at concentrations near the LoD showed no significant differences in Cq values between dilution conditions in AH or VF (Mann–Whitney U test, *P* = 1.00 for both).

Samples were submitted to the data center at Oita University, where eligibility was confirmed on the basis of the data in case report forms (CRFs), including consent or optout date, sample type, sampling date, adverse events, clinical diagnosis, laboratory test results, and treatment response. Blood or solid contamination was assessed using case report forms and macroscopic inspection, on the basis of previous validation indicating that macroscopically visible blood up to 2 μL/well does not affect PCR performance [20]; no samples met this exclusion criterion. Sample volume was measured with a pipette; samples ≤20 μL were excluded. The remaining samples were assigned an ID code, entered into a dataset, aliquoted, and blinded. Per PMDA consultation, qPCR was used as the reference standard, consistent with guidelines and owing to its high sensitivity and specificity for intraocular pathogen detection. Nucleic acid extraction using the QIAamp MinElute Virus Spin Kit (Qiagen), followed by qPCR (Nihon Techno Service), was performed as the comparator method. Direct Strip PCR (Shimadzu Corporation) was performed as the investigational method. Both methods targeted 9 pathogens: HSV-1, HSV-2, VZV, EBV, CMV, HHV-6, HTLV-1, *T gondii*, and *T pallidum.* All the procedures, including qPCR with nucleic acid extraction and Direct Strip PCR without extraction, were blinded and performed according to their respective protocols [20].

For both methods, 3-point calibration curves (1.0 × 10^2^, 1.0 × 10^4^, 1.0 × 10^6^ copies/reaction; n = 3) were measured per run (calibration curve A). Direct Strip PCR further used a cost-effective single-run (calibration curve B), measured once in advance to reflect clinical settings, with basic linearity and accuracy confirmed (data not shown). Missing or inconclusive results were to be tabulated without retesting; however, no such results were observed. When the 2 methods yielded discordant results, residual samples or DNA, if available, were reblinded and retested. To avoid overestimation, only the initial results were used for concordance analyses. The database was locked before statistical analysis. Retest findings, together with detailed CRFs—including age, sex, medical history, ophthalmic and systemic findings, treatment history, and clinical photographs—were provided solely to support review and discussion by the clinical review committee (M.M. and H.T.). On the basis of these data, the committee judged which result was more clinically plausible—Direct Strip PCR, qPCR, or both. A judgment of “both” was assigned when neither blood testing nor other supporting data provided decisive evidence, and the results of both Direct Strip PCR and qPCR were considered clinically plausible on the basis of the clinical findings. If disagreements occurred between the 2 reviewers, case discussions were planned; however, no such disagreements were observed.

### Main outcome measures

All outcomes were evaluated separately for AH and VF. The primary outcome was the positive, negative, and overall concordance rates between qPCR and Direct Strip PCR across all the pathogens, with ≥90% required in all the categories, as agreed with the PMDA. Secondary outcomes included pathogen-specific concordance rates, which were evaluated only for pathogens with ≥10 qPCR-positive samples, the minimum number prespecified to ensure sufficient statistical reliability. Safety was evaluated on the basis of adverse event occurrence.

### Statistical analysis

Concordance between both methods was tabulated, and 95% CIs were calculated by use of the Clopper–Pearson method based on a binomial distribution. Correlation coefficients were used to analyze DNA copy numbers between both methods. Adverse events were summarized according to the incidence rate. Statistical analyses were performed using SAS (version 9.4; SAS Institute), JMP Pro (version 16.0; SAS Institute.), and R (version 4.0.2; R Foundation for Statistical Computing).

## Results

### Full analysis set (FAS)/safety analysis set (SAS)

**Fig. 1:**
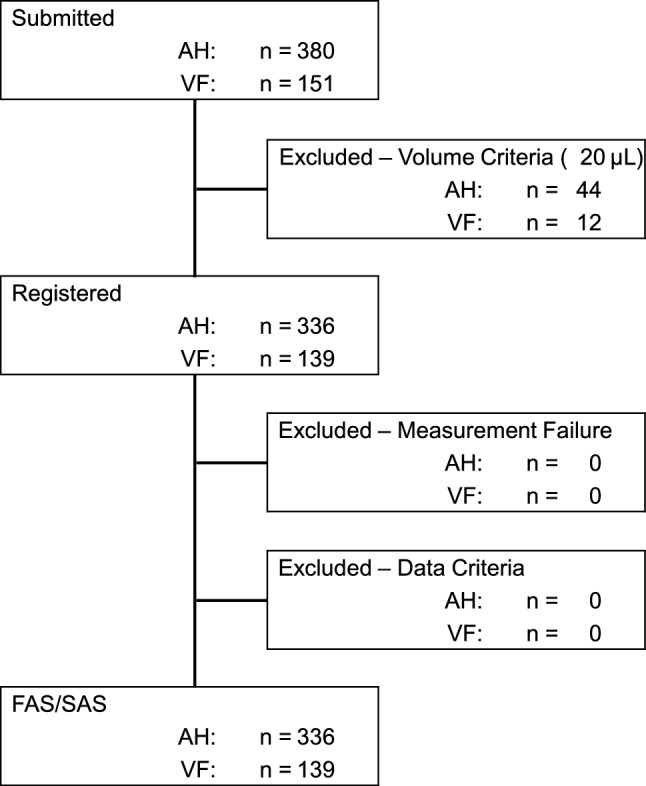
Full and safety analysis sets. A total of 531 samples were analyzed (AH: 380, VF: 151). Samples with insufficient volume (≤20 µL) were excluded, resulting in an FAS and SAS of 336 AH and 139 VF samples. No samples were excluded because of measurement failure or data criteria such as missing or inconclusive data in either method. All the outcomes were evaluated separately for AH and VF. AH indicates aqueous humor; FAS, full analysis set; SAS, safety analysis set; VF, vitreous fluid

In total, 531 samples were submitted (AH: 380; VF: 151). Samples with insufficient volume (≤20 µL) were excluded, resulting in an FAS and SAS of 336 AH and 139 VF samples. No samples were excluded owing to measurement failure or data criteria such as missing or inconclusive data in either method. All outcomes were evaluated separately for AH and VF (Fig. 1).

### Detection via qPCR and Direct Strip PCR in FAS

#### Per-pathogen-target detection (Table 1)

In AH, 212 pathogen-targets tested positive and 2807 tested negative via both Direct Strip PCR and qPCR. Among 3024 pathogen-targets (336 AH samples × 9 pathogens), 5 showed discordant results: 3 were positive only via Direct Strip PCR, and 2, only via qPCR. In VF, 73 pathogen-targets tested positive and 1173 tested negative via both methods. Among 1251 pathogen-targets (139 VF samples × 9 pathogens), 5 showed discordant results: all 5 tested positive only via Direct Strip PCR, whereas none tested positive only via qPCR.

**Table 1:** Per-pathogen target detection

		AH		VF	
		Direct Strip PCR		Direct Strip PCR	
		Positive	Negative	Positive	Negative
qPCR	Positive	212	2	73	0
	Negative	3	2807	5	1173

*AH* aqueous humor, *qPCR* quantitative polymerase chain reaction, *VF* vitreous fluid

All values represent the number of samples.

#### Per-sample detection (Table 2)

In clinical uveitis samples, mixed infections or secondary low-copy detections are occasionally observed. Table 1 shows per-pathogen-target results; Table 2 summarizes per-sample concordance across all 9 pathogens, including multiple-pathogen–positive samples. In AH, 199 samples showed positive concordance and 132 showed negative concordance across all 9 pathogens by both methods. In VF, 67 samples showed positive concordance and 67 showed negative concordance. In both AH and VF, 1-pathogen discordance was observed in 2 single-pathogen–positive samples each. In contrast, partial concordance—defined as concordance for the main causative pathogen with discordance for secondary low-copy pathogens—was observed in 3 multiple-pathogen–positive samples each.

**Table 2:** Per-sample detection

		AH		VF	
		Direct Strip PCR		Direct Strip PCR	
		Positive (partial)*	Negative	Positive (partial)*	Negative
qPCR	Positive (partial)*	199(3)	1	67(3)	0
	Negative	1	132	2	67

*AH* aqueous humor, *qPCR* quantitative polymerase chain reaction, *VF* vitreous fluid

^*^Partial concordance in multiple pathogen-positive samples.

All values represent the number of samples.

#### Detailed pathogen detection (Table 3)

Among the PCR-positive cases, CMV and VZV were the most frequently detected pathogens in AH (62 and 56 cases, respectively) and in VF (14 and 24 cases, respectively). All 9 target pathogens were detected in AH, whilst the VF samples included all except HHV-6 and *T pallidum*, owing to limited vitrectomy cases. Notably, *T pallidum* (2 cases) and *T gondii* (11 cases) were concordantly detected in AH via both methods.

**Table 3:** Detailed pathogen detection

	AH				VF			
qPCR	Positive		Negative		Positive		Negative	
Direct Strip PCR	Positive	Negative	Positive	Negative	Positive	Negative	Positive	Negative
HSV-1	32	0	0	304	1	0	0	138
HSV-2	23	0	0	313	6	0	0	133
VZV	56	0	0	280	24	0	0	115
EBV	20	1	1	314	10	0	3	126
CMV	62	1	0	273	14	0	0	125
HHV-6	1	0	0	335	0	0	0	139
HTLV-1	5	0	2	329	10	0	2	127
*Treponema pallidum*	2	0	0	334	0	0	0	139
*Toxoplasma gondii*	11	0	0	325	8	0	0	131
All pathogens	212	2	3	2807	73	0	5	1173

*AH* aqueous humor, *CMV* cytomegalovirus, *EBV* Epstein–Barr virus, *HHV-6* human herpes virus 6, *HSV1* herpes simplex virus type 1, *HSV2* herpes simplex virus type 2, *HTLV-1* human T-cell leukemia virus type 1, *qPCR* quantitative polymerase chain reaction, *VF* vitreous fluid, *VZV* varicella zoster virus

All values represent the number of samples.

#### Detailed discordant results (Table 4)

Among 4275 pathogen-targets from AH and VF, 10 discordant results between both methods were observed (0.23%), of which 8 were positive only by Direct Strip PCR. On the basis of the reblinded retesting of residual samples or DNA, when available, and the review of detailed case report forms, the clinical review committee judged the results to be clinically plausible for Direct Strip PCR in 4 cases, for qPCR in 1 case, and for both Direct Strip PCR and qPCR in 5 cases. The 1 case judged more plausible for qPCR was a low-copy CMV detection (Cq 37.06) considered the causative pathogen and was supported by a documented therapeutic response to anti-CMV treatment. The remaining 9 discordant results involved low-copy EBV or HTLV-1 near the detection threshold (Cq 36.27–38.64) and were regarded as secondary detections consistent with latent or systemic infection. Among these 9 cases, 4 Direct Strip PCR–positive results were considered clinically plausible, supported by positive blood testing for EBV or HTLV-1 and concordant clinical findings. In the remaining 5 cases, blood testing was not performed, and the results of both methods were considered clinically plausible given the clinical course.

**Table 4:** Detailed discordant results

Type	Pathogens	qPCR				Direct Strip PCR				More clinically plausible result*
		Initial	Cq	Retest	Cq	Initial	Cq	Retest	Cq	(judged by clinical review committee)
AH	CMV	Positive	37.06	Negative		Negative		Negative		qPCR
	EBV	Positive	36.68	Negative		Negative		Positive	37.34	Both
	EBV	Negative		Negative		Positive	38.62	–		Both
	HTLV-1	Negative		Negative		Positive	38.18	Positive	36.07	Direct Strip PCR
	HTLV-1	Negative		Negative		Positive	38.01	–		Direct Strip PCR
VF	EBV	Negative		Negative		Positive	36.62	–		Direct Strip PCR
	EBV	Negative		Negative		Positive	36.27	–		Direct Strip PCR
	EBV	Negative		Negative		Positive	37.54	–		Both
	HTLV-1	Negative		Negative		Positive	38.64	–		Both
	HTLV-1	Negative		Negative		Positive	36.28	–		Both

*AH* aqueous humor, *CMV* cytomegalovirus, *Cq* quantification cycle, *EBV* Epstein–Barr virus, *HTLV-1* human T-cell leukemia virus type 1, *VF* vitreous fluid

^*^On the basis of the reblinded retesting of residual samples or DNA, when available, and review of detailed case report forms, the clinical review committee judged the results to be clinically plausible for Direct Strip PCR in 4 cases, for qPCR in 1 case, and for both Direct Strip PCR and qPCR in 5 cases.

### Primary outcome measures

#### Percent positive agreement (PPA), percent negative agreement (PNA), and percent overall agreement (POA) between both methods across all pathogens (Table 5)

In AH, the PPA, PNA, and POA were 98.0% (95% CI: 95.0–99.5), 99.2% (95% CI: 95.9–100.0), and 98.5% (95% CI: 96.6–99.5), respectively. In VF, these values were 95.7% (95% CI: 88.0–99.1), 97.1% (95% CI: 89.9–99.6), and 96.4% (95% CI: 91.8–98.8), respectively. All the values for both AH and VF exceeded 90%, meeting the predefined performance criteria agreed upon with the PMDA.

**Table 5:** Percentage positive, percentage negative, and percentage overall agreements

Type	Pathogens	qPCR-positive	PPA (%)	95% CI	PNA (%)		POA (%)	95% CI
AH	HSV-1	32*	100.0	(89.1–100.0)	100.0	(98.8–100.0)	100.0	(98.9–100.0)
	HSV-2	23*	100.0	(85.2–100.0)	100.0	(98.8–100.0)	100.0	(98.9–100.0)
	VZV	56*	100.0	(93.6–100.0)	100.0	(98.7–100.0)	100.0	(98.9–100.0)
	EBV	21*	95.2	(76.2–99.9)	99.7	(98.2–100.0)	99.4	(97.9–99.9)
	CMV	63*	98.4	(91.5–100.0)	100.0	(98.7–100.0)	99.7	(98.4–100.0)
	HHV-6	1	100.0	(2.5–100.0)	100.0	(98.9–100.0)	100.0	(98.9–100.0)
	HTLV-1	5	100.0	(47.8–100.0)	99.4	(97.8–99.9)	99.4	(97.9–99.9)
	*Treponema pallidum*	2	100.0	(15.8–100.0)	100.0	(98.9–100.0)	100.0	(98.9–100.0)
	*Toxoplasma gondii*	11	100.0	(71.5–100.0)	100.0	(98.9–100.0)	100.0	(98.9–100.0)
	All Pathogens	214	98.0	(95.0–99.5)	99.2	(95.9–100.0)	98.5	(96.6–99.5)
VF	HSV-1	1	100.0	(2.5–100.0)	100.0	(97.4–100.0)	100.0	(97.4–100.0)
	HSV-2	6	100.0	(54.1–100.0)	100.0	(97.3–100.0)	100.0	(97.4–100.0)
	VZV	24*	100.0	(85.8–100.0)	100.0	(96.8–100.0)	100.0	(97.4–100.0)
	EBV	10*	100.0	(69.2–100.0)	97.7	(93.4–99.5)	97.8	(93.8–99.6)
	CMV	14*	100.0	(76.8–100.0)	100.0	(97.1–100.0)	100.0	(97.4–100.0)
	HHV-6	0	–		100.0	(97.4–100.0)	100.0	(97.4–100.0)
	HTLV-1	10*	100.0	(69.2–100.0)	98.4	(94.5–99.8)	98.6	(94.9–99.8)
	*T pallidum*	0	–		100.0	(97.4–100.0)	100.0	(97.4–100.0)
	*T gondii*	8	100.0	(63.1–100.0)	100.0	(97.2–100.0)	100.0	(97.4–100.0)
	All pathogens	73	95.7	(88.0–99.1)	97.1	(89.9–99.6)	96.4	(91.8–98.8)

*AH* aqueous humor, *CMV* cytomegalovirus, *EBV* Epstein–Barr virus, *HHV-6* human herpes virus 6, *HSV1* herpes simplex virus type 1, *HSV2* herpes simplex virus type 2, *HTLV-1* human T-cell leukemia virus type 1, *qPCR* quantitative polymerase chain reaction, *PPA* percentage positive agreement, *PNA* percentage negative agreement, *POA* percentage overall agreement, *VF* vitreous fluid, *VZV* varicella zoster virus

^*^The PPA, PNA, and POA between both methods were evaluated for each pathogen with ≥10 qPCR-positive samples.

### Secondary outcomes

#### PPA, PNA, and POA between both methods for each pathogen with ≥10 qPCR-positive samples (Table 5)

The PPA, PNA, and POA between both methods were evaluated for each pathogen with ≥10 qPCR-positive samples, a statistically sufficient number preagreed with the PMDA. For HSV-1, HSV-2, VZV, EBV, CMV, and *T gondii* in AH, PPA was 95.2%–100.0%; PNA, 99.7%–100.0%; and POA, 99.4%–100.0%. For VZV, EBV, CMV, and HTLV-1 in VF, PPA was 100.0%; PNA, 99.7%–100.0%; and POA, 97.8%–100.0%.

#### Cq values and DNA copy numbers using qPCR and Direct Strip PCR (Tables 6 and 7)

The Cq values and DNA copy numbers obtained using qPCR and Direct Strip PCR in AH and VF are summarized in Tables 6 and 7, respectively. Across all the pathogens in AH, the median Cq values were comparable between qPCR (30.5) and Direct Strip PCR (30.7), with similar ranges (16.8–39.6 for qPCR; 16.9–41.4 for Direct Strip PCR). The median DNA copy numbers were 2.42 × 10^5^ (qPCR), 4.90 × 10^5^ (Direct Strip PCR, curve A), and 4.22 × 10^5^ (curve B), with comparable minimums and maximums (qPCR: 4.73 × 10^2^–2.73 × 10^9^; curve A: 3.63 × 10^2^–2.00 × 10^9^; curve B: 5.17 × 10^2^–2.36 × 10^9^). In VF, the median Cq values were 27.6 (qPCR) and 28.3 (Direct Strip PCR), with similar ranges (qPCR: 18.8–39.8; Direct Strip PCR: 18.3–40.5). The median DNA copy numbers were 1.82 × 10^6^ (qPCR), 1.74 × 10^6^ (Direct Strip PCR, curve A), and 2.28 × 10^6^ (curve B), with minimum and maximum values of 3.04 × 10^2^–7.11 × 10^8^ (qPCR), 6.58 × 10^2^–1.79 × 10^9^ (curve A), and 7.09 × 10^2^–1.02 × 10^9^ (curve B).

**Table 6:** Cq values and DNA copy numbers in aqueous humor

Type	Pathogens	Method/Curves*		Cq			Copy numbers (copies/mL)*		
				Median	Min	Max	Median	Mini	Max
AH	HSV-1	qPCR	A	31.9	25.6	38.4	1.38 × 10^5^	2.57 × 10^3^	3.82 × 10^6^
		Direct Strip PCR	A	31.8	26.5	38.7	3.48 × 10^5^	3.22 × 10^3^	6.64 × 10^6^
			B				3.46 × 10^5^	4.23 × 10^3^	8.96 × 10^6^
	HSV-2	qPCR	A	31.2	25.6	34.9	5.08 × 10^5^	3.31 × 10^4^	1.80 × 10^7^
		Direct Strip PCR	A	32.5	25.5	35.5	1.94 × 10^6^	2.42 × 10^5^	7.81 × 10^7^
			B				1.18 × 10^6^	2.24 × 10^5^	6.54 × 10^7^
	VZV	qPCR	A	26.0	16.8	39.6	5.27 × 10^6^	8.96 × 10^2^	2.73 × 10^9^
		Direct Strip PCR	A	26.6	16.9	41.4	6.39 × 10^6^	3.63 × 10^2^	2.00 × 10^9^
			B				5.40 × 10^6^	5.17 × 10^2^	2.36 × 10^9^
	EBV	qPCR	A	32.3	23.7	37.3	8.37 × 10^4^	3.59 × 10^3^	2.37 × 10^7^
		Direct Strip PCR	A	32.5	25.4	39.5	8.53 × 10^4^	8.63 × 10^2^	1.28 × 10^7^
			B				8.24 × 10^4^	6.98 × 10^2^	1.11 × 10^7^
	CMV	qPCR	A	29.5	20.1	37.1	2.98 × 10^5^	3.09 × 10^3^	1.74 × 10^8^
		Direct Strip PCR	A	30.3	20.4	36.1	3.08 × 10^5^	3.71 × 10^3^	2.21 × 10^8^
			B				3.10 × 10^5^	7.27 × 10^3^	1.95 × 10^8^
	HHV-6	qPCR	A	36.0	36.0	36.0	3.43 × 10^3^	3.43 × 10^3^	3.43 × 10^3^
		Direct Strip PCR	A	38.7	38.7	38.7	9.62 × 10^2^	9.62 × 10^2^	9.62 × 10^2^
			B				2.99 × 10^3^	2.99 × 10^3^	2.99 × 10^3^
	HTLV-1	qPCR	A	36.4	35.1	37.8	4.66 × 10^3^	1.04 × 10^3^	1.16 × 10^4^
		Direct Strip PCR	A	37.9	34.8	39.2	3.94 × 10^3^	1.14 × 10^3^	5.24 × 10^4^
			B				4.23 × 10^3^	1.75 × 10^3^	3.51 × 10^4^
	*Treponema pallidum*	qPCR	A	31.8	31.1	32.6	1.05 × 10^5^	6.50 × 10^4^	1.44 × 10^5^
		Direct Strip PCR	A	32.0	29.7	34.3	2.36 × 10^5^	1.29 × 10^4^	4.59 × 10^5^
			B				1.90 × 10^5^	1.95 × 10^4^	3.60 × 10^5^
	*Toxoplasma gondii*	qPCR	A	33.8	30.4	39.1	1.14 × 10^4^	4.73 × 10^2^	1.45 × 10^5^
		Direct Strip PCR	A	34.3	29.2	39.9	6.47 × 10^4^	1.62 × 10^3^	1.68 × 10^6^
			B				7.54 × 10^4^	1.73 × 10^3^	2.26 × 10^6^
	All pathogens	qPCR	A	30.5	16.8	39.6	2.42 × 10^5^	4.73 × 10^2^	2.73 × 10^9^
		Direct Strip PCR	A	30.7	16.9	41.4	4.9 × 10^5^	3.63 × 10^2^	2.00 × 10^9^
			B				4.22 × 10^5^	5.17 × 10^2^	2.36 × 10^9^

*AH* aqueous humor, *CMV* cytomegalovirus, *EBV* Epstein–Barr virus, *HHV-6* human herpes virus 6, *HSV1* herpes simplex virus type 1, *HSV2* herpes simplex virus type 2, *HTLV-1* human T-cell leukemia virus type 1, *qPCR* quantitative polymerase chain reaction, *VF* vitreous fluid, *VZV* varicella zoster virus

^*^Three-point calibration curves (1.0 × 10^2^, 1.0 × 10^4^, and 1.0 × 10^6^ copies/reaction; n = 3) were measured per run as calibration curve A. Additionally, Direct Strip PCR used a cost-effective single-run calibration curve B measured separately to reflect clinical settings.

**Table 7:** Cq values and DNA copy numbers in vitreous fluid

Type	Pathogens	Method/Curves*		Cq			Copy numbers (copies/mL)*		
				Median	Min	Max	Median	Mini	Max
VF	HSV-1	qPCR	A	25.2	25.2	25.2	1.71 × 10^7^	1.71 × 10^7^	1.71 × 10^7^
		Direct Strip PCR	A	27.4	27.4	27.4	1.38 × 10^7^	1.38 × 10^7^	1.38 × 10^7^
			B				5.22 × 10^6^	5.22 × 10^6^	5.22 × 10^6^
	HSV-2	qPCR	A	26.0	22.1	38.5	1.27 × 10^7^	5.28 × 10^3^	1.46 × 10^8^
		Direct Strip PCR	A	27.2	24.8	36.9	3.40 × 10^7^	8.91 × 10^4^	1.79 × 10^8^
			B				2.52 × 10^7^	9.69 × 10^4^	9.85 × 10^7^
	VZV	qPCR	A	22.1	18.8	35.6	1.03 × 10^8^	1.13 × 10^4^	7.11 × 10^8^
		Direct Strip PCR	A	21.9	18.3	35.3	1.72 × 10^8^	2.09 × 10^4^	1.79 × 10^9^
			B				1.10 × 10^8^	2.44 × 10^4^	1.02 × 10^9^
	EBV	qPCR	A	32.3	20.7	36.5	7.00 × 10^4^	5.83 × 10^3^	1.27 × 10^8^
		Direct Strip PCR	A	35.7	22.1	37.7	1.28 × 10^4^	2.61 × 10^3^	1.18 × 10^8^
			B				9.53 × 10^3^	2.50 × 10^3^	1.03 × 10^8^
	CMV	qPCR	A	26.8	18.8	33.5	2.51 × 10^6^	1.67 × 10^4^	5.04 × 10^8^
		Direct Strip PCR	A	26.8	18.4	34.8	4.19 × 10^6^	1.47 × 10^4^	8.45 × 10^8^
			B				3.99 × 10^6^	1.76 × 10^4^	7.19 × 10^8^
	HTLV-1	qPCR	A	35.0	32.1	37.0	7.61 × 10^3^	1.45 × 10^3^	4.38 × 10^4^
		Direct Strip PCR	A	36.2	29.3	40.5	1.49 × 10^4^	6.58 × 10^2^	1.62 × 10^6^
			B				1.34 × 10^4^	7.09 × 10^2^	1.46 × 10^6^
	*Toxoplasma gondii*	qPCR	A	30.2	26.8	39.8	2.03 × 10^5^	3.04 × 10^2^	2.02 × 10^6^
		Direct Strip PCR	A	31.1	27.1	38.6	6.25 × 10^5^	2.79 × 10^3^	6.70 × 10^6^
			B				6.20 × 10^5^	4.24 × 10^3^	9.10 × 10^6^
	All pathogens	qPCR	A	27.6	18.8	39.8	1.82 × 10^6^	3.04 × 10^2^	7.11 × 10^8^
		Direct Strip PCR	A	28.3	18.3	40.5	1.74 × 10^6^	6.58 × 10^2^	1.79 × 10^9^
			B				2.28 × 10^6^	7.09 × 10^2^	1.02 × 10^9^

*AH* aqueous humor, *CMV* cytomegalovirus, *Cq* quantification cycle, *EBV* Epstein–Barr virus, *HSV1* herpes simplex virus type 1, *HSV2* herpes simplex virus type 2, *HTLV-1* human T-cell leukemia virus type 1, *qPCR* quantitative polymerase chain reaction, *VF* vitreous fluid, *VZV* varicella zoster virus

^*^Three-point calibration curves (1.0 × 10^2^, 1.0 × 10^4^, and 1.0 × 10^6^ copies/reaction; n = 3) were measured per run as calibration curve A. Additionally, Direct Strip PCR used a cost-effective single-run calibration curve B measured separately to reflect clinical settings.

#### Correlation of DNA copy numbers between qPCR and Direct Strip PCR across all pathogens

**Fig. 2:**
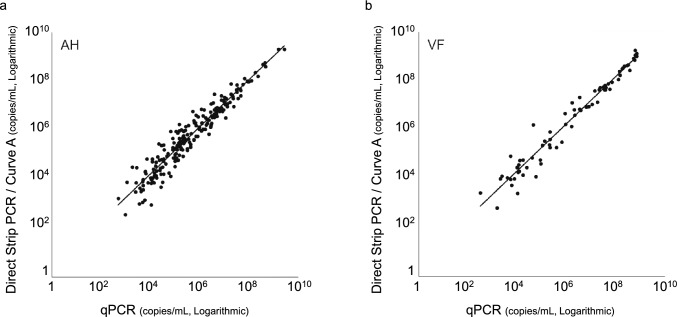
Correlation of DNA copy numbers between qPCR and Direct Strip PCR. Strong correlations in DNA copy numbers between both methods were observed in **a** AH (r = 0.957) and **b** VF (r = 0.948), on the basis of calibration curve A *(P* <.001 for both). AH indicates aqueous humor; PCR, polymerase chain reaction; qPCR, quantitative real-time PCR; VF, vitreous fluid

Strong correlations of DNA copy numbers between both methods were observed in AH (r = 0.957 and 0.996) and in VF (r = 0.948 and 0.951), on the basis of calibration curves A and B, with all correlations being significant (*P* <.001). Figure 2 shows the results with calibration curve A, the standard per-run curve.

#### Correlation of DNA copy numbers between qPCR and Direct Strip PCR for each pathogen with ≥10 qPCR-positive samples

The correlation coefficients between both methods were evaluated for pathogens with ≥10 qPCR-positive samples. For HSV-1, HSV-2, VZV, EBV, CMV, and *T gondii* in AH, the median coefficient was 0.877 (95% CI: 0.659–0.969); for VZV, EBV, CMV, and HTLV-1 in VF, it was 0.967 (95% CI: 0.929–1.000). All correlations were significant, with *P* <.001.

#### Correlation of DNA copy numbers between calibration curves A and B (Table 8 and Figure 3)

Strong correlations were observed between calibration curves A and B in AH (r = 0.943) and VF (r = 0.981; *P* <.001 for both).

**Table 8:** Correlation of DNA copy numbers across all pathogens

Type	Method/Curves*	Correlation coefficient	*P* value	Standard deviation		
AH	qPCR/Curve A	0.957	<.001	2.00 × 10^8^	/	2.19 × 10^8^
	qPCR/Curve B	0.996	<.001	1.84 × 10^8^	/	2.19 × 10^8^
	Curve A/B	0.943	<.001	2.00 × 10^8^	/	1.84 × 10^8^
VF	qPCR/Curve A	0.948	<.001	3.38 × 10^8^	/	1.78 × 10^8^
	qPCR/Curves B	0.951	<.001	2.13 × 10^8^	/	1.78 × 10^8^
	Curves A/B	0.981	<.001	3.38 × 10^8^	/	2.13 × 10^8^

*AH* aqueous humor, *qPCR* quantitative polymerase chain reaction, *VF* vitreous fluid

^*^Calibration curve A is generated per measurement using 3 simultaneous positive controls, whereas calibration curve B is a cost-effective representative curve (n = 3) from independently measured controls for clinical practice. Standard deviations are shown for qPCR/ Direct Strip PCR, respectively.

**Fig. 3:**
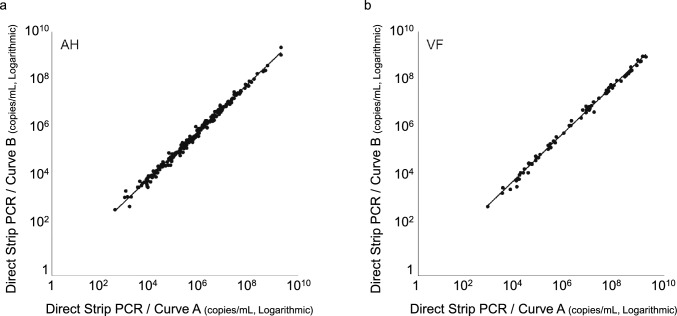
Correlation of DNA copy numbers between calibration curves A and B. Strong correlations between calibration curves A and B were observed for a AH (r = 0.943) and b VF (r = 0.981), with *P* <.001 for both. AH indicates aqueous humor; VF, vitreous fluid

#### Cq values and DNA copy numbers for each disease (Table 9)

The Cq values and DNA copy numbers are presented for each disease, with final clinical diagnoses based on the CRFs in 285 Direct Strip PCR/qPCR-positive cases. Direct Strip PCR quantified DNA across a wide dynamic range: 3.04 × 10^2^–2.73 × 10^9^ copies/mL (median 3.39 × 10^5^), with Cq values of 16.79–39.75 (median 29.86). All 9 target pathogens, including the rare *T pallidum*, were identified in patient ocular fluids, supporting the clinical relevance of the Direct Strip PCR target optimization. HTLV-1, EBV, and HHV-6 may be detected in ocular fluids secondary to systemic or latent infection; thus, causality requires exclusion of other causes of disease and careful clinical correlation. Final diagnoses were classified as either causative or secondary on the basis of the CRFs. By pathogen, in AH, CMV (62 cases: anterior uveitis/corneal endotheliitis, CMV retinitis) was the most frequently detected, followed by VZV (56 cases: ARN, herpetic anterior uveitis). In VF, VZV (24 cases: ARN) and CMV (14 cases: CMV retinitis) were also frequently detected. AH samples from CMV retinitis were often collected during intravitreal ganciclovir injections (off-label use) to assess disease activity and treatment response on the basis of DNA copy numbers. By disease, ARN was most frequent, with HSV-1 (26 AH/1 VF), HSV-2 (23 AH/6 VF), and VZV (30 AH/24 VF), highlighting the need for rapid PCR using both AH (for early diagnosis at the initial visit) and surgical residual VF (for activity assessment at the site of disease onset). The median DNA copy numbers were higher in VF than in AH (*P* = .020) and in VZV than in HSV (*P* <.001); however, these comparisons are based on samples from different patients (AH: HSV 5.08 × 10^5^, VZV 1.84 × 10⁷; VF: HSV 1.71 × 10⁷, VZV 1.03 × 10^8^ copies/mL; Mann–Whitney U test).

**Table 9:** Cq values and DNA copy numbers by Direct Strip PCR for each disease

Type	Pathogens	Final clinical diagnosis	Direct Strip PCR/	Direct Strip PCR					
			qPCR-positive	Cq			Copy numbers (copies/mL)*		
				Median	Min	Max	Median	Mini	Max
AH	HSV-1	Herpetic anterior uveitis	26	32.88	25.59	38.37	8.39 × 10^4^	2.57 × 10^3^	3.82 × 10^6^
		Acute retinal necrosis	6	29.90	25.99	34.83	8.11 × 10^5^	2.64 × 10^4^	2.92 × 10^6^
	HSV-2	Acute retinal necrosis	23	31.23	25.63	34.91	5.08 × 10^5^	3.31 × 10^4^	1.80 × 10^7^
	VZV	Acute retinal necrosis	25	24.75	18.13	32.87	1.84 × 10^7^	7.75 × 10^4^	1.50 × 10^9^
		Herpetic anterior uveitis	30	26.99	16.79	39.64	2.57 × 10^6^	8.96 × 10^2^	2.73 × 10^9^
		PORN	1	30.75	–	–	1.19 × 10^5^	–	–
	EBV	EBV-positive uveitis	12	31.14	23.71	34.13	2.07 × 10^5^	2.59 × 10^4^	2.37 × 10^7^
		Secondary**	8	35.37	29.01	37.28	1.80 × 10^4^	3.59 × 10^3^	6.93 × 10^5^
	CMV	Corneal endotheliitis/AU	36	31.69	21.69	36.65	7.51 × 10^4^	4.52 × 10^3^	3.08 × 10^7^
		CMV retinitis	26	25.98	20.13	32.88	2.78 × 10^6^	3.25 × 10^4^	1.74 × 10^8^
	HHV-6	HHV-6-positive uveitis	1	36.44	–	–	4.96 × 10^3^	–	–
	HTLV-1	HTLV-1 associated uveitis	4	36.80	35.11	37.77	3.69 × 10^3^	1.04 × 10^3^	1.16 × 10^4^
		Secondary**	1	32.25	–	–	1.42 × 10^5^	–	–
	*Treponema pallidum*	Syphilitic uveitis	2	31.81	31.05	32.57	1.05 × 10^5^	6.50 × 10^4^	1.44 × 10^5^
	*Toxoplasma gondii*	Ocular toxoplasmosis	11	33.81	30.40	39.13	1.14 × 10^4^	4.73 × 10^2^	1.45 × 10^5^
VF	HSV-1	Acute retinal necrosis	1	25.16	–	–	1.71 × 10^7^	–	–
	HSV-2	Acute retinal necrosis	6	26.00	22.11	38.54	1.27 × 10^7^	5.28 × 10^3^	1.46 × 10^8^
	VZV	Acute retinal necrosis	24	22.12	18.83	35.57	1.03 × 10^8^	1.13 × 10^4^	7.11 × 10^8^
	EBV	EBV-positive uveitis	8	30.94	20.68	36.47	1.42 × 10^5^	5.83 × 10^3^	1.27 × 10^8^
		Secondary**	2	34.27	32.93	35.60	3.11 × 10^4^	7.41 × 10^3^	5.47 × 10^4^
	CMV	CMV retinitis	14	26.78	18.75	33.51	2.51 × 10^6^	1.67 × 10^4^	5.04 × 10^8^
	HTLV-1	HTLV-1 associated uveitis	8	35.00	32.13	37.01	7.81 × 10^3^	1.98 × 10^3^	4.38 × 10^4^
		Secondary**	2	35.60	34.22	36.98	5.77 × 10^3^	1.45 × 10^3^	1.01 × 10^4^
	*T gondii*	Ocular toxoplasmosis	8	30.21	26.79	39.75	2.03 × 10^5^	3.04 × 10^2^	2.02 × 10^6^

*AH* aqueous humor, *AU* anterior uveitis, *CMV* cytomegalovirus, *Cq* quantification cycle, *EBV* Epstein–Barr virus, *HSV1* herpes simplex virus type 1, *HSV2* herpes simplex virus type 2, *HTLV-1* human T-cell leukemia virus type 1, *PORN* progressive outer retinal necrosis, *qPCR* quantitative polymerase chain reaction, *VF* vitreous fluid, *VZV* varicella zoster virus

^*^Three-point calibration curves (1.0 × 10^2^, 1.0 × 10^4^, and 1.0 × 10^6^ copies/reaction; n = 3) were measured per run as calibration curve A. Additionally, Direct Strip PCR used a cost-effective single-run calibration curve B measured separately to reflect clinical settings.

^**^Secondary detections of EBV, HTLV-1, and HHV-6 are pathogens known to establish latent or systemic infection in the human body.

### Safety analysis

No adverse events related to the sample collection procedures (anterior chamber paracentesis or vitrectomy) were reported in the SAS.

### Additional analysis

In this study, the samples were diluted, unlike in routine practice, to address limited volume and to evaluate performance near the LoD. To assess whether dilution altered the results under these stringent conditions, per-sample detections (Supplemental Table 1), agreement metrics (PPA, PNA, POA; Supplemental Table 2), and copy-number correlations (Supplemental Table 3) were evaluated by dilution status. No significant differences in concordance were observed between the undiluted and diluted samples (Fisher exact test, all *P* = .32–1.00); quantitative correlations were preserved (r = 0.956–0.987).

## Discussion

We analyzed 475 ocular fluid samples after standard exclusions, with no cases omitted for test failure or missing data, confirming the robustness and safety of the sample collection. Our findings also surpassed the PMDA criteria, with >30 qPCR-positive cases across all the pathogens and >50 qPCR-negative cases for all 9 pathogens, ensuring solid statistical power. Despite the rarity of infectious uveitis, the successful sample collection from numerous facilities across Japan reflects significant clinical demand for insurance coverage of this diagnostic modality. No adverse events were reported during sampling, confirming the safety of anterior chamber paracentesis. Most AH samples were collected using the AH pipette (Scimen Design), which features a short 30-G needle to minimize lens injury and a straw-like syringe enabling 1-handed operation [24]. AH sampling is an established, insurance-covered procedure, including for interleukin-10/interleukin-6 cytokine testing in intraocular lymphoma. These safety findings further support the regulatory approval of Direct Strip PCR.

For the primary outcome, Direct Strip PCR showed high concordance with qPCR across all the pathogens (95.7%–98.5%), exceeding the 90% PMDA threshold. This high concordance was also observed for each pathogen, with agreement rates ≥95.2%. Furthermore, consistently high PNA across all the 9 major pathogens supports its utility in ruling out infections—an essential step before initiating anti-inflammatory treatment, per the JOIS guidelines on the use of the tumor necrosis factor [16]. Misdiagnosis and glucocorticoid monotherapy can cause rapid pathogen proliferation and poor outcomes, especially without pathogen-specific therapy [25].

In this study, all 9 target pathogens were detected in the ocular fluids, covering the main causes of infectious uveitis reported in Japanese epidemiology [1], listed in order of frequency: herpetic iritis (HSV-1/2, VZV, CMV), ARN (HSV-1/2, VZV), CMV retinitis, HTLV-1–associated uveitis, ocular toxoplasmosis, syphilis-associated uveitis, EBV-associated uveitis, and other viral uveitis. Herpetic iritis is the most common infectious uveitis, having risen from fifth place in 2002 [26] and 2009 [27] to third overall in the 2016 survey [1]. Furthermore, with CMV as its major cause, the proportion of hepatic iritis had increased from 27% to 48% in the 2016 survey. ARN ranks second, with all cases PCR-diagnosed and 76.1% VZV-positive. Consistent with these epidemiologic data, CMV and VZV were also the most frequently detected pathogens in both AH and VF. For context, FilmArray and Verigene offer rapid multiplex PCR for systemic infections but require large sample volumes and are unsuitable for ocular fluids. The nCounter system enables high-throughput multiplex detection but needs extraction and has long turnaround times. Metagenomic NGS detects all pathogens without previous sequence knowledge but is costly and lab-bound [28]. Direct Strip PCR is distinct in enabling 32-min detection of the 9 major uveitis pathogens from only 20 µL of ocular fluid, with high qPCR concordance.

An extremely low discordance rate was observed, with only 5 discordant targets each in AH and VF, totaling 10 of the 4275 pathogen-targets tested. These discordant results had high Cq values (36.27–38.64), most representing secondary detections of EBV or HTLV-1—both of which, along with HHV-6, are pathogens known to cause latent or systemic infection. Additionally, no discordance was observed in the causative pathogens, except for 1 low-copy CMV. Eight targets were positive only via Direct Strip PCR, supporting its advantage in avoiding DNA loss during qPCR extraction (70.6% recovery) [20].

Intraocular viral load is useful for diagnosing the causative pathogen, disease activity monitoring, treatment response assessment, and prognostication in infectious uveitis. In VZV-AU, the viral load correlates with iris atrophy and flare [6], whilst in CMV-AU and corneal endotheliitis, it correlates with increased intraocular pressure, corneal endothelial damage, and recurrence, and viral load parallels clinical improvement [7, 29–31]. In HSV/VZV-ARN, the viral load correlates with retinal detachment, extent of necrosis, and visual prognosis [5, 32]. The Cq values showed similar ranges between qPCR (16.8–39.8) and Direct Strip PCR (16.9–41.4), both within their measurement range. Strong correlations in DNA copy numbers were observed between the 2 methods in both AH and VF (r = 0.951–0.996, *P *<.001), suggesting Direct Strip PCR use for qualitative diagnosis of the causative pathogen and for quantitative monitoring, such as assessment of disease activity or treatment response. In ARN, the viral load typically declines through a plateau, logarithmic decrease, and negativity over a mean of 56 days; a high initial load or <50% reduction at 2 weeks predicts poor visual outcome [33]. Direct Strip PCR is the standard diagnostic test in the JOIS and AMED-led Japan-Acute Retinal Necrosis Registry [34], used for both initial causative pathogen diagnosis by distinguishing HSV-1/2 from severe VZV and for quantitative monitoring of disease activity at the initial visit, surgery, and intravenous or oral therapy completion. In CMV retinitis, CMV antibody testing has limited utility (80%–90% seroprevalence), and the blood-based C7-HRP antigen assay poorly reflects intraocular disease. By contrast, Direct Strip PCR enables pathogen monitoring using AH collected during ganciclovir intravitreal injections (off-label), supporting diagnosis and treatment evaluation.

Our most practical finding was the strong correlation between calibration curve A (per-run) and cost-saving curve B (single-run), with r = 0.943–0.981 (*P* <.001), supporting the clinical applicability of curve B. Additional advantages over previous multiplex PCR kits [35] include integrated internal controls—GAPDH (50-copy spike-in, PCR reaction control), TBP (human inflammatory cell marker, sample quality control), and negative wells—to ensure assay validity without external calibration; a solid-phase format eliminating DNA extraction or liquid handling and reducing user error and contamination risk; true quantitative capability for all targets; ease of use and rapid workflow requiring 1 min of preparation without nucleic-acid extraction; multiplexing from 20 µL samples with the quantification of 9 pathogens; and high clinical stability and reproducibility with minimal centralized-testing lot variation (coefficient of variation [CV] 0.2%–1.7% [18]) and comparably minimal variability across sites, instruments, and operators (CV 1.4% for experts and 1.8% for beginners [20]). This was in clear contrast to qPCR, which showed substantially greater operator-dependent variability (CV 4.7% for experts and 63.6% for beginners), confirming the assay’s high robustness and generalizability—which may be even greater with IVD/QMS-grade manufacturing—and supporting its suitability for both central and point-of-care laboratories. Although dilution showed no effect in this study, it is not recommended because high variability was observed among beginners [20]; moreover, excessive freeze–thaw cycles and prolonged room-temperature storage should be avoided to ensure the accurate representation of intraocular pathogen load.

With the nationwide adoption of Direct Strip PCR for infectious uveitis, the number of tests sent directly from clinics to outsourced diagnostic laboratories has recently surpassed those from university hospitals conducting Advanced Medical Care, which were the primary testing route during the era of complex quantitative PCR assays. Performing PCR testing at the pretreatment stage during clinic visits is an optimal approach for causative pathogen diagnosis. For example, in cytomegalovirus corneal endotheliitis, the time to diagnosis decreased from 3.5 years to 3 months following the widespread adoption of multiplex PCR for infectious uveitis. Earlier diagnosis was associated with improved clinical outcomes, including better final visual acuity and corneal endothelial cell counts, and reduction in glaucoma surgeries [36].

This study has some limitations. Although HHV-6 and *T pallidum* were not detected in VF, they were confirmed in AH, and all the other pathogens were detected in both. Therefore, detection of these pathogens in VF is considered feasible. Moreover, not all the pathogens responsible for infectious uveitis are included in the Direct Strip PCR. *Toxocara* and *Mycobacterium tuberculosis* are excluded to avoid false negatives, as their immune-mediated disease mechanisms result in rare detection in ocular fluid [20, 37], especially in developed countries. Clinicians should consider systemic tests, such as blood tests and chest X-rays, when these excluded infections are suspected. For rubella in Fuchs uveitis syndrome, the antibody index is available [38], and RT-PCR is labor-intensive; therefore, it is not currently included in our kit but remains a future challenge. For global implementation of this multiplex PCR kit, laboratory, health system, regulatory, and cost-related factors must be considered. Finally, although agreement with qPCR was required as the primary endpoint under PMDA criteria for IVD clinical performance studies, it does not represent the true diagnostic accuracy. Because PCR detection does not necessarily indicate the causative pathogen, interpreting results in isolation may lead to misclassification; thus, PCR findings must be integrated with clinical information, including medical history, ophthalmic and systemic findings, and treatment history, to ensure appropriate clinical interpretation.

In conclusion, this multicenter study showed that Direct Strip PCR is a highly reliable, safe, and clinically applicable diagnostic test for infectious uveitis. It achieved high concordance with qPCR across all target pathogens, with PPA, PNA, and POA consistently exceeding PMDA-defined thresholds, yielding quantitative results that correlated with qPCR results. All major uveitis pathogens relevant to epidemiology [1] were included, and the assay proved effective for both diagnosis of the causative pathogen and quantitative monitoring. While standardized multiplex PCR platforms remain scarce worldwide [35], its solid-phase, extraction-free, per-run calibration-free format enables the global dissemination of multiplex PCR and may thus address this gap and promote adoption of PCR-based diagnosis. As regulatory submissions advance in Japan and internationally, including Brazil, this Japan-originated innovation could improve the visual prognosis for infectious uveitis worldwide.

## Direct Strip PCR Project Group (*Participating study sites, affiliations at submission)

**Table:** 

1. Kobe City Eye Hospital* Sunao Sugita, Shinichirou Itoh, Ayumi Hono
2. Tokyo Medical and Dental University*(Institute of Science Tokyo) Hiroshi Takase, Chiharu Hayashi
3. Gunma University* Daisuke Todokoro, Mayumi Hosogai, Hideo Akiyama, Kazuma Saito, Shunsuke Tokui, Yosuke Arai, Yoko Nakatani
4. University of Tsukuba* Yumi Hasegawa, Fumiki Okamoto, Tetsuro Oshika, Sujin Hoshi
5. University of Miyazaki* Yasuhiro Ikeda, Naohiro Sugita, Takako Hidaka, Makiko Mori, Natsuki Kajihara, Miyuki Imazato, Masataka Ishizu, Michiko Kuroki, Chinami Tamura, Midori Horinouchi, Naoki Toyama, Shunpei Hayashi, Erika Hirose, Chiaki Arai, Yusuke Okuno, Ikki Mitsutome, Akira Hayashida, Go Mawatari, Keisuke Nakayama
6. Kyoto Prefectural University of Medicine* Chie Sotozono, Kenji Nagata, Noriko Koizumi, Hideto Deguchi, Takanori Aoki, Hideki Fukuoka, Tomoko Horikiri
7. Kansai Medical University* Kaoru Araki-Sasaki, Hisanori Imai, Shimpei Oba, Hidetsugu Mori
8. Jichi Medical University Saitama Medical Center* Suguru Nakagawa, Toshikatsu Kaburaki, Akihiro Kakehashi, Katsuaki Tanaka, Yoshinari Saima, Yugo Hiranuma, Yukihiko Nakajima, Yuka Kondo, Yoshitaka Orio, Hidenobu Asami, Ryo Taguchi
9. Hiroshima University* Yoshiaki Kiuchi, Taiichiro Chikama, Yousuke Harada, Ryotaro Toda, Atsuhiko Fukuto, Koichiro Shinji, Tomona Hiyama, Kaori Mitoma
10. Kyushu University* Koh-Hei Sonoda, Nobuyo Yawata, Eiichi Hasegawa, Yusuke Murakami, Keijiro Ishikawa, Shoji Notomi, Sayaka Myojin, Fumiyo Morikawa, Yuka Matsutani
11. Tohoku University* Takehiro Hariya, Toru Nakazawa, Shunji Yokokura, Wataru Kobayashi, Kazuki Hashimoto, Masaaki Yoshida, Takuya Kiryama
12. Fukuoka University* Eiichi Uchio, Tomoko Tsukahara-Kawamura
13. Oita University* Toshiaki Kubota, Atsunobu Takeda, Kenichi Kimoto, Takako Nakamuro, Katsuhiko Yokoyama, Ryoko Oki, Daichi Kishi, Yoshihiro Noda, Koichiro Tamura, Shunsuke Kano, Toru Adachi, Shota Hino, Hiroyuki Yatsuka, Maho Itotani, Shiho Fukui, Jun Funatsu, Ayako Okita, Mami Otsuka, Takaaki Otsuka, Yoshiki Sato, Tomoya Ishibe, Toru Kageyama
14. Ehime University* Hidenori Inoue, Koji Toriyama, Atsushi Shiraishi, Yuko Hara, Yuri Sakane, Yuki Takezawa, Yukako Hiramatsu, Wakako Ikegawa
15. Kobe University* Sentaro Kusuhara, Wataru Matsumiya, Kyung Woo Kim, Rei Sotani
16. Japan Community Health Care Organization Osaka Hospital* Nobuyuki Ohguro, Hisashi Mashimo
17. Kochi University* Ken Fukuda, Tamaki Sumi, Takashi Nishiuchi, Asami Nakahira, Yusaku Miura, Tatsuma Kishimoto, Waka Ishida, Kayo Sugiura, Minori Kumada, Misa Masaoka, Tomoka Mizobuchi, Atsuki Fukushima; Yoshie Nishida (Department of Clinical Laboratory)
18. Tokushima University* Ryoji Yanai, Mariko Egawa
19. Yodogawa Christian Hospital* Kei Nakai, Akira Tanigawa
20. University of Fukui* Masakazu Morioka, Takeshi Tomomatsu, Tomoe Aoki, Masaru Inatani, Tamami Yamamoto
21. University of Toyama* Atsushi Hayashi, Shuichiro Yanagisawa, Tomoko Ueda, Akio Miyakoshi, Tomoko Nakamura, Masaaki Ishida
22. Tokyo Medical University* Yoshihiko Usui, Hiroshi Goto, Naoyuki Yamakawa, Takeshi Kezuka
23. Miyata Eye Hospital* Ryohei Nejima, Yosai Mori, Toshihiro Sakisaka, Shigefumi Takahashi, Kouji Ueda, Eriko Kanaya, Yuto Inafuku, Takahiro Hisai, Tomohiro Yokogawa, Naoto Kuwabara, Takuya Iwasaki, Manabu Mochizuki, Kazunori Miyata
24. Hokkaido University* Kenichi Namba, Susumu Ishida, Daiju Iwata, You Ogino, Keitaro Hase, Kayo Suzuki
25. Kyorin University* Annabelle A. Okada, Hiroshi Keino, Makiko Nakayama, Shoko Saito, Katsuya Nagahori, Akito Hirakata
26. Yokohama City University* Nobuhisa Mizuki, Masaki Takeuchi, Yuki Mizuki, Shigeru Kawano
27. Tottori University* Dai Miyazaki, Yoshitugu Inoue, Tomoko Haruki, Ayumi Koyama
28. Beppu Medical Center* Kunihiro Kiyosaki
29. Asahikawa Medical University* Reiko Kinouchi, Tsugiaki Utsunomiya, Takayuki Satoh, Masatoshi Sado, Taiji Nagaoka
30. Yamaguchi University Ryoji Yanai, Kazuhiro Kimura, Naoyuki Yamada, Tomohiko Nagai, Sho-Hei Uchi, Nanako Iwamoto, Makoto Hatano
31. National Center for Global Health and Medicine, Japan Institute for Health Security Shigeko Yashiro, Miyuki Naghara, Harumi Fukushima, Yuuka Yamamoto, Hiroshi Murata
32. The University of Tokyo Rie Tanaka, Takashi Miyai, Tetsuya Toyono, Yukako Taketani, Takashi Ono, Toshikatsu Kaburaki
33. Shimane University Aika Tsutsui, Masaki Tanito, Kaoru Manabe, Katsue Imamachi; Chikashi Matsuda (Clinical Laboratory Division)
34. Toho University Yuichi Hori, Koji Kakisu, Takashi Suzuki
35. Ishizuchi Eye Clinic Takashi Suzuki
36. Jichi Medical University Toshikatsu Kaburaki, Meri Watanabe, Kanako Annoura
37. Saitama Medical University Kei Shinoda; Takuya Maeda, Yuta Orihara (Department of Clinical Laboratory Medicine)
38. Nagoya University Hiroaki Ushida, Koji Nishiguchi, Ayana Suzumura, Yoshito Koyanagi, Hiroki Kaneko, Norie Nonobe, Satoshi Okado, Taro Kominami, Hikaru Ota, Keigo Natsume, Kenya Yuki, Yasuki Ito; Kanae Okada (Department of Medical Technique, Clinical Laboratory)
39. The University of Osaka Kazuichi Maruyama, Kohji Nishida, Noriyasu Hashida, Kazunobu Asao, Satoko Fujimoto; Jun Kawasaki, Yumi Sasaki (BIKEN)
40. Kindai University Hiroshi Eguchi, Fumika Hotta, Chiharu Iwahashi, Masuo Sakamoto
41. Health Sciences University of Hokkaido Nobuyoshi Kitaichi, Yukihiro Horie
42. Juntendo University Urayasu Hospital Nobuyuki Ebihara, Riyu Ikari
43. Teikyo University Yuji Inoue, Ayano Enomoto
44. Tokyo Dental College Ichikawa General Hospital Takefumi Yamaguchi, Daisuke Tomita, Yukari Taniguchi, Taiyoh Shijoh, Yuma Kayama
45. Juntendo University Shintaro Nakao, Toshiaki Hirakata; Yoko Tabe, Koji Tsuchiya, Yoshie Hosaka (Department of Clinical Laboratory)
46. Miyata Eye Hospital Tokyo Clinic Manabu Mochizuki, Hiroshi Takase, Yukiko Terada
47. Kanazawa Medical University Tsuyoshi Mito
48. Fujita Health University Yasuki Ito, Tadashi Mizuguchi, Koichiro Yata, Miyuki Takahashi, Ryoko Nomura, Yui Nariai, Kohsuke Sekido, Masashi Kimata, Daisuke Kato, Shunken Kato, Koji Ueoka, Akito Torii, Momo Shimizu
49. Niigata University Takeo Fukuchi, Tadamichi Akagi, Hiroko Terashima, Ryu Iikawa, Simon Kurosawa, Naota Kobayashi Niigata Prefecture Yoshida Hospital Keiko Suzuki
50. Aichi Medical University Motohiro Kamei, Atsuya Miki, Kyoko Fujita, Shinjiro Kouno, Kotaro Tsuboi, Keita Baba, Yuichiro Ishida, Kento Hirai, Ai Shibata, Keiko Yamamoto, Hikari Ono, Masakazu Oiwa, Kazuyuki Majima, Akane Sobue, Mizuki Hamada, Taro Muratani, Tomohiro Tanaka, Ayako Ito, Tomoko Kuroda, Taiki Kodo, Akiko Jinno, Ai Shibata
51. Kyoto University Kenji Ishihara, Yuki Muraoka, Takanori Kameda, Hanako Ikeda, Kenji Suda, Masahiro Miyake, Shogo Numa, Yuki Mori, Eri Nakano, Naoko Ueda
52. Tsukazaki Hospital Atsuki Fukushima
53. Wakayama Medical University Hiroki Iwanishi, Takayoshi Sumioka, Yukihisa Takada, Shingo Yasuda, Kousuke Nishi, Ai Matsushita, Kazuki Imai, Chika Yamamoto, Yudai Yamaguchi
54. Okayama University Yuki Morizane, Mio Hosokawa, Miyuki Fujiwara, Yusuke Shiode, Ryo Matoba, Shin Morisawa, Hiroshi Matsumae, Tetsuro Morita, Hiroya Kindo
55. Kawasaki Medical School Hiroyuki Kamao
56. Kurume University Shigeo Yoshida, Kensuke Sasaki, Yoshiki Kojima, Chikako Taguchi
57. University of Occupational and Environmental Health, Japan Hiroyuki Kondo, Tatsuo Nagata, Itsuka Matsushita, Kazuma Oku, Madoka Taniguchi
58. Kyushu Medical Center Shintaro Nakao
59. Saga University Hiroshi Enaida, Hiroaki Sakai, Isao Nakao
60. Nagasaki University Masafumi Uematsu, Akio Oishi, Sugao Miyagi, Yuki Hirata, Shiori Harada
61. University of the Ryukyus Hideki Koizumi, Yamauchi Yukihide, Nobuhiro Terao, Naoya Imanaga, Ayano Oshiro; Yoshino Tokashiki (Division of Clinical Laboratory and Blood Transfusion)

## Supplementary Information

Below is the link to the electronic supplementary material.

Supplementary file1 (DOCX 21 KB)
