# Supplementary material for: Clinical performance of the multiplex solid-phase “Direct Strip PCR” for infectious uveitis: a multicenter diagnostic accuracy study
Source: Jpn J Ophthalmol. 2026 Apr 2;70(4):966–82. doi: 10.1007/s10384-026-01343-2 (PMC13452761; doi:10.1007/s10384-026-01343-2)
Supplement: Supplementary file 1 [file 10384_2026_1343_MOESM1_ESM.docx]

**Supplemental Table 1** Per-sample detection by dilution status

| Dilution status |  |  | AH |  | VF |  |
| --- | --- | --- | --- | --- | --- | --- |
|  |  |  | Direct Strip PCR | | Direct Strip PCR | |
|  |  |  | Positive  (partial)* | Negative | Positive  (partial)* | Negative |
| Undiluted | qPCR | Positive (partial)* | 77(2) | 0 | 37(2) | 0 |
|  |  | Negative | 0 | 90 | 1 | 47 |
| Diluted** | qPCR | Positive (partial)* | 122(1) | 1 | 30(1) | 0 |
|  |  | Negative | 1 | 42 | 1 | 20 |

*AH* aqueous humor, *qPCR* quantitative polymerase chain reaction, *VF* vitreous fluid

*Partial concordance in multiple pathogen-positive samples.

**All 9 pathogens in AH and 7 in VF were detected in the undiluted samples; in the diluted samples (median AH 1.8-fold [1.1–5.0], VF 2.6-fold [1.1–5.0]), only HHV-6 in AH and HSV-1 in VF were absent.

All values represent the number of samples.

**Supplemental Table 2** Percentage positive, percentage negative, and percentage overall agreements by dilution status

| Type | Dilution status | Pathogens | qPCR-positive |  | PPA (%) | 95% CI | | | | | PNA (%) |  | | | | | POA (%) | 95% CI | | | | |
| --- | --- | --- | --- | --- | --- | --- | --- | --- | --- | --- | --- | --- | --- | --- | --- | --- | --- | --- | --- | --- | --- | --- |
| AH | Undiluted | All Pathogens | 85 |  | 97.5 | ( | 91.2 | ‐ | 99.7 | ) | 100 | ( | 96.0 | ‐ | 100.0 | ) | 98.8 | ( | 95.8 | ‐ | 99.9 | ) |
|  | Diluted | All Pathogens | 129 |  | 98.4 | ( | 94.3 | ‐ | 99.8 | ) | 97.7 | ( | 87.7 | ‐ | 99.9 | ) | 98.2 | ( | 94.8 | ‐ | 99.6 | ) |
| VF | Undiluted | All Pathogens | 41 |  | 94.9 | ( | 82.7 | ‐ | 99.4 | ) | 97.9 | ( | 88.9 | ‐ | 99.9 | ) | 96.6 | ( | 90.3 | ‐ | 99.3 | ) |
|  | Diluted | All Pathogens | 32 |  | 96.8 | ( | 83.3 | ‐ | 99.9 | ) | 95.2 | ( | 76.2 | ‐ | 99.9 | ) | 96.2 | ( | 86.8 | ‐ | 99.5 | ) |

*AH* aqueous humor, *qPCR* quantitative polymerase chain reaction, *PPA* percentage positive agreement, *PNA* percentage negative agreement, *POA* percentage overall agreemen, *VF* vitreous fluid

*The PPA, PNA, and POA between the 2 methods for all pathogens were evaluated by dilution status. No significant differences in concordance were observed between the undiluted and the diluted samples (Fisher exact test; all *P* = 0.32–1.00).

**Supplemental Table 3** Correlation of DNA copy numbers across all pathogens by dilution status

| Type | Dilution status | Method/Curves* | Correlation coefficient | *P* value | Standard deviation | | |
| --- | --- | --- | --- | --- | --- | --- | --- |
| AH | Undiluted | qPCR/Curve A | 0.983 | <.001 | 5.59 × 10^7^ | / | 5.09 × 10^7^ |
|  | Diluted | qPCR/Curve A | 0.956 | <.001 | 2.70 × 10^8^ | / | 2.47 × 10^8^ |
| VF | Undiluted | qPCR/Curve A | 0.937 | <.001 | 1.66 × 10^8^ | / | 3.59 × 10^8^ |
|  | Diluted | qPCR/Curve A | 0.987 | <.001 | 1.95 × 10^8^ | / | 3.14 × 10^8^ |

*AH* aqueous humor, *qPCR* quantitative polymerase chain reaction, *VF* vitreous fluid

*Calibration curve A is generated per measurement using 3 simultaneous positive controls (n = 3). Standard deviations are shown for qPCR / Direct Strip PCR, respectively.
